# The Case for Community Health Innovation Networks*

**DOI:** 10.1145/3209811.3212705

**Published:** 2018-06-20

**Authors:** Isaac Holeman, Ari Johnson, Kassoum Kayentao, Youssouf Keita, Stephen Odindo, Caroline Whidden

**Affiliations:** Medic Mobile Seattle USA isaac@medicmobile.org; University of California at San Francisco and Muso USAajohnson@musohealth.org; Muso Bamako Mali kkayentao@musohealth.org; Muso Bamako Mali ykeita@musohealth.org; Living Goods Nairobi Kenya sodindo@livinggoods.org; Muso Bamako Mali cwhidden@musohealth.org

**Keywords:** community health workers, digital health, global health, human-centered design, ICT4D, implementation science, mHealth

## Abstract

This commentary1 outlines the rationale for building community health innovation networks in settings of poverty and high burdens of disease. These networks integrate deep research expertise and sustained implementation infrastructure, with the aim of streamlining the design, building and scale up of evidence-based technical innovations for community health. Drawing on our experiences establishing such networks in Mali and Kenya, we discuss the importance of: 1) sustaining operational capacity to strengthen health systems; 2) being strategic about how we embed design research within ongoing implementation efforts; and 3) institutional partnerships that invest in infrastructure to support a series of studies and innovation efforts over time. Without claiming to offer any ‘quick and easy’ solutions, we argue that this approach has real potential to address the gap between research and practice in technical innovation for global health and sustainable development.

**CCS CONCEPTS:**

• Human-centered computing

## 1 INTRODUCTION

Sustainable Development Goal 3’s call to action on good health and well-being highlights remarkable progress in recent decades, against a backdrop of striking inequalities. Maternal mortality has fallen by nearly 50% since 1990, yet it remains 14 times higher in developing regions than in developed regions [1]. A remarkable 17,000 fewer children die each day than in 1990, but every year over 5 million children still die before their fifth birthday [2]. Children born into poverty are nearly twice as likely to die before age five as children of wealthier families [1]. These signs of progress and ongoing challenges hinge in no small part on access to basic health services. As the World Health Organization’s recent report on universal health coverage observed, more than half the world’s population still lacks access to basic health care [3]. In the poor and hard-to-reach communities where our organizations work, patients must overcome geographic, financial, gender, and infrastructural barriers on the path to care. People who visit clinics face multiple indirect costs, including out-of-pocket travel expenses and the loss of as much as a full day’s wages. As a result, easily curable illnesses such as malaria, pneumonia and diarrheal diseases are often treated late or not at all.

Research spanning several decades shows that CHW-led health care systems can address these challenges [4]. For example, an evaluation of Living Goods’ work in Uganda documented a 27% reduction in child mortality through the provision of data-driven, doorstep care [5]. In Mali, Muso documented a reduction in child mortality from 154 deaths per 1000 live births in 2008 to just seven per 1000 by 2015, after the roll-out of a model emphasizing proactive house visits, user fee removal, and primary care capacity building [6]. Yet recent studies of national-scale programs have shown that inadequately trained, professionalized or supervised CHWs may have little or no impact on child mortality [4,7-9]. The problem is that CHWs are often unpaid, poorly equipped, and not integrated into a robust, accessible primary care system. Routine operations can make or break efforts to grow beyond a promising study and deliver sustained impact at scale. While there is no easy way to eliminate these challenges, using technology to support new models of data-driven community health shows great promise. New technologies and systems innovations with potential to accelerate health impacts have been documented extensively in the ICT4D and digital health literatures. However, in too many cases they have proven no more scalable than the health programs that they aim to streamline or strengthen. Many efforts fail to design new tools in a human-centered manner that addresses the contextual complexities of delivering care in challenging settings. Others embrace a more human-centered design practice, yet lack the operational expertise or practitioner networks to bring promising innovations to scale.

Responding to these challenges, this commentary outlines our rationale for establishing community health innovation networks in settings of poverty and high burdens of disease. Such networks involve long term partnerships across multiple organizations with complementary areas of focus, to integrate deep technical, research, delivery, and contextual expertise. Drawing on our experiences establishing such networks in Mali and Kenya, we discuss:

1. Making pragmatic investments in a strong health system;
2. Maintaining routine health information systems;
3. Establishing a shared delivery innovation agenda that addresses drivers of the burden of disease;
4. Building capacity for rigorous, multidisciplinary research;
5. Leveraging built-in pathways to scale;
6. Open sourcing a community health toolkit including code, user guides and technical resources to support scale up;
7. Prioritizing long term institutional partnerships.

This approach expects a great deal more than collecting data and writing publications; it is arduous and not without risks for the narrowly-focused academic. Nevertheless, we believe it holds potential to significantly streamline the design, building and scale up of evidence-based innovations for community health.

## 2 HEALTH SYSTEM STRENGTHENING AND IMPLEMENTATION RESEARCH

As a multidisciplinary field that draws heavily on medicine and public health, global health research is typically informed by insights about trends and drivers of the global burden of disease. While some of these insights are region or disease-specific, many now recognize that a growing proportion of the global burden of morbidity and mortality can be attributed to conditions for which treatments are well known to be effective and affordable. While much of the world is benefiting from an era of increasingly high-tech medicine, consistently delivering even the most basic health services in settings of poverty and high burdens of disease remains remarkably challenging.

Because of this “implementation bottleneck,” the most striking opportunities to advance human health and well being may now lie in equipping program implementers to make basic services more accessible and equitable. To this end, World Bank president Jim Kim, physician-anthropologist Paul Farmer and acclaimed strategy researcher Michael Porter [10] call for a bold new agenda for global health delivery research. Using the related terms of implementation research and implementation science, a growing number of funding schemes, new journals, and degree-granting programs have emerged to address this challenge [11]. One important direction for implementation research has been to reinvigorate the decades-old health system strengthening agenda. For example, there is widespread medical consensus that the Ebola outbreak which devastated several West African countries from 2013 to 2016 would never have spread in settings with more robust public health infrastructure. The death toll was particularly high among health workers, who in many places ran out of personal protective equipment like rubber gloves and lacked facilities to dispose safely of medical waste (Ebola patients produce up to 40x more medical waste than other patients, due to extensive vomiting and diarrhea) [12]. Tech companies such as Google, Amazon and Ericsson and techie charities such as the Paul G. Allen Family Foundation attempted to support the Ebola response by donating thousands of smartphones. Yet these contributions were roundly critiqued as tone deaf in a situation where health workers were dying in droves for lack of access to rubber gloves. In other words, these efforts were critiqued for having exhibited a technology or innovation fetish that was out of sync with local health systems strengthening priorities [13]. The “Build Back Better” slogan that gained popularity among Ebola responders generally emphasized the importance of strengthening the whole health system rather than treating Ebola as an isolated event [14].

Another important opportunity for implementation research has to do with systems innovations that reimagine how we organize and deliver care. For example, six leading community health organizations recently compared their operational practices and identified eight common features that characterize the design of advanced community health systems [15] (see Box 1). Highly pragmatic in nature, these practices are delivery or systems innovations in the sense that they remain debated or not widely embraced by community health programs globally. Researchintensive community health organizations are thus refining and evaluating them, with the aim of establishing broader medical consensus concerning their impact on health outcomes, their affordability, and the practical specifics of supporting them in remote, low-infrastructure communities.

Mobile technologies have featured prominently in global health implementation research. As one World Health Organization report on mobile health observed, “mHealth has the potential to transform the face of health service delivery across the globe” [16]. However, failure to move from promising pilot to implementation at scale is now so common that many in the global health technology field bemoan a widespread “pilotitis” [17,18]. This high failure rate has prompted growing recognition that mHealth interventions are deeply complex [19,20]. Their outcomes typically rely on changes in health worker or patient practices, the tools evolve constantly and they are often implemented in organizational contexts characterized by poor coordination, ambiguity and rapid change.

□Box 1: Key Features of Strong Community Health Systems

1. Accredited: The health knowledge and competencies of CHWs are assessed prior to practicing; CHWs must meet a minimum standard before carrying out their work.
2. Accessible: To improve accessibility, timeliness, and equity of care, point-of-care user fees should be avoided when possible.
3. Proactive: For active disease surveillance, CHWs go door-to-door looking for sick patients and providing training on how to identify danger signs and quickly contact a CHW.
4. Continuously Trained: CHWs are trained using modular delivery or other types of in-service learning. Continuing medical education is not only available to but required of CHWs.
5. Supported by a Dedicated Supervisor: On a frequent and regular basis, CHWs benefit from a dedicated supervisor who assesses patient experience and provides 1-on-1 coaching.
6. Paid: CHWs are compensated financially at a competitive rate relative to the respective market.
7. Strong Health System: CHW deployment is accompanied by investments to increase the capacity, accessibility, and quality of the primary care facilities and providers to which CHWs link, including pharmacy management.
8. Data Feedback Loops: CHWs report all data to public-sector monitoring and evaluation systems and data get used by those who collected it to improve programs and CHW performance.

For this reason, a growing number of global health and development organizations are exploring how they might design new tools in a more human-centered manner that addresses the contextual complexities of delivering care in challenging settings. The Principles for Digital Development consensus statement now urges practitioners to “design with the user,” “employ a ‘systems’ approach to design,” “develop projects in an incremental and iterative manner,” and “work across sector silos to create coordinated and more holistic approaches” [21]. These principles have been widely endorsed by major global health institutions, including several United Nations agencies, the United States Agency for International Development, and the Bill and Melinda Gates Foundation.

To date, however, widespread lack of clarity remains about how to put these design principles into practice [21,22]. One recent review recognized ongoing tensions related to the fact that human-centered design and the global health field have different underlying conceptual models and terminology [23]. Few global health and development organizations have cultivated in-house expertise in doing ethnographic design-oriented fieldwork, involving users in the design process, and documenting rich insights in ways that are actionable for product developers. This kind of design competency is present at all major tech companies and many academic research labs in the fields of ICT4D, human computer interaction and human-centered design. However, these institutions often lack an operational model that can extend the R&D process from promising innovation to implementation at scale in the world’s most challenging settings. In the following section we consider how these labs might help to bridge this gap by embedding their work more strategically within community health innovation efforts.

## 3 STRATEGICALLY EMBEDDED HUMAN-CENTERED DESIGN RESEARCH

While many ICT4D research efforts undertake iterative design-build-test cycles driven by real-world applications, they vary greatly with respect to how they frame the “real world” and their engagement with it. A recent survey of ICT4D researchers concluded that, “while ICT4D researchers are interested in influencing both practice and policy, they are less inclined toward the activities that would make this happen” [24]. As researchers affiliated with practitioner organizations, we see this as one of the more striking challenges facing the ICT4D community. In our view, simply spending more time on after-the-fact dissemination activities would, at best, only address part of the problem. Instead, we would like to offer a more fundamental reappraisal of how we might embed ICT4D research in the world of practice.

Take for example a recent series of papers on the use of digital tools for personalized performance feedback among community health workers in India [25,26]. The investigators undertook an extensive, participatory design process and evaluated their intervention’s effects through a rigorous randomized trial. They found that CHWs in the intervention group performed 20% more house visits than peers in the control group. Yet performance in the control group dropped more than 20% during the course of the study, and even intervention group performance dropped nominally during the evaluation period. The authors attribute the overall decline in performance to underlying changes in the community health system rather than to their intervention—the face-to-face supervision activities that had been routine at baseline were discontinued during much of their study period.

In many respects this study is a best-in-class example of rigorous ICT4D research with potential for real world impact. Yet from a community health perspective, the actual decline in CHW performance makes it difficult to view this intervention as a ready-to-scale, unmitigated success story. In particular, it remains unclear how we should generalize the study findings to community health systems that do sustain face-to-face supervision—can we still expect a 20% performance boost, or would the digital tool be relatively more redundant and provide less additional benefit beyond face-to-face supervision? This is not to say that this study was a failure, but rather that we regard this kind of evidence as an important yet incomplete contribution to science. For the design researcher embedded in the health systems strengthening and delivery innovation discourses described above, there is a clear need for further studies to redesign and evaluate the technology and the underlying model of community health care in tandem. This point resonates with the critique that sociotechnical and sociomaterial design researchers have long made of artificially separating technical and social concerns [22,27]. The authors of this particular study seem acutely aware of these issues, but among ICT4D researchers in general it would be more common to simply frame the underlying health system issues as beyond the researcher’s control, highlight the promise of the technology, and move on to the next project.

This case underscores a point made previously by Anokwa et al [28]: strategic decisions about where to undertake design projects, who to involve as design partners and which health or development issues to frame as design challenges are often made implicitly, before the application of documented design methods. Given that these choices have a major bearing on the ultimate impacts of ICT4D interventions, we would argue that they should feature more prominently in our research reports. We would like to see such discussion in the literature lead to new kinds of partnerships and operational models that better integrate ICT4D research and global health research in pragmatic community health innovation efforts. The following section outlines our current efforts to address these challenges.

## 4 HEALTH INNOVATION NETWORKS

Without wishing to preclude alternative approaches, our aim in this section is to highlight distinguishing features of our strategy for establishing community health innovation networks.

1. Making pragmatic investments in a strong health system
  Focusing narrowly on high tech innovations in communities where people are dying for lack of trained health workers or stock outs of basic medical supplies is likely to be ineffectual, and perhaps even problematic. For this reason, we approach research with a ‘care-first’ scientific agenda. This means involving practitioners where appropriate and refashioning research timelines and budgets to support the routine functioning of the health system. Such pragmatic investments good for patients, and they also improve access to data, surface new insights and make for better and more relevant science.
2. Maintaining routine community health information systems
  Maintaining a robust health information systems is an important part of health systems strengthening. Once such systems are in place, each R&D project can focus on testing a specific software feature set or diagnostic device without needing to build basic data systems at the beginning of every study.
3. Integrating the design of technology and health systems
  New technologies can only reach as far as the health systems that deliver them. For a delivery innovation agenda and human-centered design process to reflect this insight, it is important to reimagine technology, care workflows, and health system management practices together. We are focusing on innovations that can scale within the advanced community health systems described above, rather than focusing on how technology can fill gaps in systems that lack basic human resources.
4. Building capacity for rigorous, multidisciplinary research
  Design researchers and health researchers are typically trained in different methods and conceptual frameworks. They publish in different places, and espouse different notions of scientific rigor. It takes time and mutual effort to develop a shared language and practical ways of benefiting from each others’ work [29]. In our experience, human-centered design researchers have much to offer with user research, iteration, prototyping practices and the building of new tools. Health researchers can situate innovation projects in relation to a health systems strengthening and implementation research agenda, driven by insights about the local burden of disease. They can also establish monitoring systems and field experiments to evaluate impacts on a health system’s coverage, quality, speed and equity.
5. Leveraging built-in pathways to scale
  Innovation projects typically begin with pilots involving fewer than 200 health workers. Leveraging a built-in pathway to scale entails situating such pilots within larger implementer networks. For example, Living Goods established an innovation network with under 200 CHWs within a broader network of over 9,000 CHWs. Muso’s innovation network exists within a decade-old Ministry of Health partnership and global CHW policy agenda, supported not only by researchers but also by capacity building and policy staff. These arrangements enable delivery innovations to benefit, from the earliest stages of exploratory research, from practitioners’ practical expertise in scaling innovations.
6. Open sourcing a shared community health toolkit
  Our shared community health toolkit includes open source code, disease models and algorithms, user guides and technical resources, and de-identified community health datasets. When shared as public goods, these resources enable a quick start for new projects and support scale up of promising innovations.
7. Prioritizing long term institutional partnerships
  Sustained institutional partnership is necessary for the practical reason that it would typically be infeasible for any one lab, practitioner organization or ministry of health to maintain all of this infrastructure alone. While each network establishes shared infrastructure among complementary organizations in a specific place, sharing resources across innovation networks may also make these efforts more sustainable and effective [30], particularly when it comes to repeatedly testing promising innovations in different health systems.

## 5 CONCLUSIONS

The gap between research and practice is a serious concern for many ICT4D researchers. This paper offers pragmatic suggestions for addressing this gap through the formation of community health innovation networks. Establishing this kind of network requires substantial ongoing investment of time and resources, yet the benefits are clear when the resulting infrastructure lasts long enough to support many delivery innovation and research initiatives over time. Strategic choices about whether or how researchers make investments in shared infrastructure have a major bearing on the ultimate impact of ICT4D interventions. It is our hope that this paper will prompt further discussion of how new partnerships and operational models might integrate research and practice, not only in community health but more broadly to amplify the benefits of computing for sustainable development.
